# Impact of Spiritual or Faith-Based Interventions in Inpatient Psychiatric Treatment: A Descriptive Systematic Review

**DOI:** 10.1192/j.eurpsy.2026.10990

**Published:** 2026-07-31

**Authors:** L. M. Kebede, N. Chandrasekaran, M. Zamora, H. Raai

**Affiliations:** Psychiatry and Behavioral Health, SBH Health System, New York City, United States

## Abstract

**Introduction:**

Spiritual and religious dimensions of health are often overlooked in psychiatric treatment, despite growing evidence suggesting their relevance in recovery. This study systematically reviews the literature on spiritual or faith-based interventions in inpatient psychiatric settings to evaluate their impact on psychological and emotional outcomes.

**Objectives:**

To evaluate the role and impact of spiritual and faith-based interventions on psychological and emotional outcomes in adult inpatient psychiatric settings.

To synthesize existing empirical evidence on the feasibility, acceptability, and therapeutic value of integrating spirituality into psychiatric care.

**Methods:**

A descriptive systematic review was conducted using PubMed, covering studies published in English between 2010 and 2024. Eligible studies included adult participants (≥18 years) in inpatient psychiatric or residential care who received spiritual interventions such as chaplaincy services, pastoral counseling, group prayer, or spiritually integrated psychotherapy. Outcomes assessed included symptom reduction, coping, therapeutic alliance, patient satisfaction, and spiritual/emotional well-being. Both qualitative and quantitative empirical studies were included. A narrative synthesis approach was used due to heterogeneity in interventions and outcome measures.

**Results:**

Out of 52 records identified, 10 studies met inclusion criteria. Interventions were implemented in diverse psychiatric settings and addressed a wide range of mental health conditions including serious mental illness and co-occurring substance use disorders. Common modalities included group sessions centered on meaning-making, hope, forgiveness, and existential reflection. Several models (e.g., ACTing Spiritually, SPIRIT protocol) demonstrated utility in enhancing coping skills and emotional regulation. Qualitative findings emphasized improvements in emotional expression, interpersonal connection, and reduced isolation. Quantitative results indicated a substantial patient demand for spiritual support, even among non-religious individuals. However, most studies lacked standardized outcome measures or control groups.

**Conclusions:**

Spiritual and faith-based interventions are feasible, acceptable, and appear to complement psychiatric care in inpatient settings. They contribute to patient-centered recovery by addressing spiritual needs and fostering emotional resilience. Despite encouraging findings, further high-quality research is needed to clarify best practices, efficacy, and strategies for integration into interdisciplinary care teams.

**Disclosure of Interest:**

None Declared

